# Identification of Intrinsic Drug Resistance and Its Biomarkers in High-Throughput Pharmacogenomic and CRISPR Screens

**DOI:** 10.1016/j.patter.2020.100065

**Published:** 2020-07-02

**Authors:** Iñigo Ayestaran, Ana Galhoz, Elmar Spiegel, Ben Sidders, Jonathan R. Dry, Frank Dondelinger, Andreas Bender, Ultan McDermott, Francesco Iorio, Michael P. Menden

**Affiliations:** 1 Institute of Computational Biology, Helmholtz Zentrum München GmbH—German Research Center for Environmental Health, Neuherberg 85764, Germany; 2 CRUK Cambridge Centre Early Detection Programme, Department of Oncology, University of Cambridge, Cambridge, UK; 3 Department of Biology, Ludwig-Maximilians University Munich, Martinsried 82152, Germany; 4 Research and Early Development, Oncology, AstraZeneca, Cambridge CB4 0WG, UK; 5 Research and Early Development, Oncology, AstraZeneca, Boston, MA 02451, USA; 6 Tempus Labs, Boston, MA, USA; 7 Centre for Health Informatics, Computation and Statistics, Lancaster Medical School, Lancaster University, Lancaster LA1 4YW, UK; 8 Centre for Molecular Informatics, Department of Chemistry, University of Cambridge, Lensfield Road, Cambridge CB2 1EW, UK; 9 Wellcome Sanger Institute, Wellcome Genome Campus, Hinxton, Cambridge CB10 1RQ, UK; 10 Human Technopole, Palazzo Italia Via Cristina Belgioioso 171, Milano 20157, Italy; 11 German Centre for Diabetes Research (DZD e.V.), Neuherberg 85764, Germany

**Keywords:** precision medicine, cancer, drug resistance, early drug discovery, biostatistics, biomarker discovery, drug high-throughput screens, cancer cell lines, CRISPR, drug combinations

## Abstract

High-throughput drug screens in cancer cell lines test compounds at low concentrations, thereby enabling the identification of drug-sensitivity biomarkers, while resistance biomarkers remain underexplored. Dissecting meaningful drug responses at high concentrations is challenging due to cytotoxicity, i.e., off-target effects, thus limiting resistance biomarker discovery to frequently mutated cancer genes. To address this, we interrogate subpopulations carrying sensitivity biomarkers and consecutively investigate unexpectedly resistant (UNRES) cell lines for unique genetic alterations that may drive resistance. By analyzing the GDSC and CTRP datasets, we find 53 and 35 UNRES cases, respectively. For 24 and 28 of them, we highlight putative resistance biomarkers. We find clinically relevant cases such as EGFR^T790M^ mutation in NCI-H1975 or PTEN loss in NCI-H1650 cells, in lung adenocarcinoma treated with EGFR inhibitors. Interrogating the underpinnings of drug resistance with publicly available CRISPR phenotypic assays assists in prioritizing resistance drivers, offering hypotheses for drug combinations.

## Highlights

• Presenting a strategy for identifying rare drug-resistance markers in cancer cells
• Effectively stratifying drug resistance from cytotoxicity in pharmacology screens
• Generating hypotheses for resistance mechanisms underlying certain biomarkers
• CRISPR screens support resistance hypotheses and shed light on cancer vulnerabilities

## The Bigger Picture

Cancer drug resistance is the major challenge of modern oncology. Identifying resistance and its biomarkers will empower the next generation of precision medicines. High-throughput pharmacology screens in cancer cell lines have successfully identified drug-sensitivity biomarkers, but drug-resistance biomarkers are underexplored. Intrinsic drug-resistance events are often rare and experimentally indistinguishable from cytotoxicity or artifacts without prior knowledge. To address this, we investigate cell-line populations sensitized to a drug treatment (i.e., carrying established sensitivity biomarkers) and characterize those cell lines that do not respond as expected. We highlight unique genetic features harbored by these cell lines and confirm their linkage to drug resistance using CRISPR gene essentiality data. Our analysis and results pave the way for enhanced precision medicine, guide further CRISPR screens, and identify potential drug combinations to tackle resistance.

## Graphical Abstract

**Figure:**
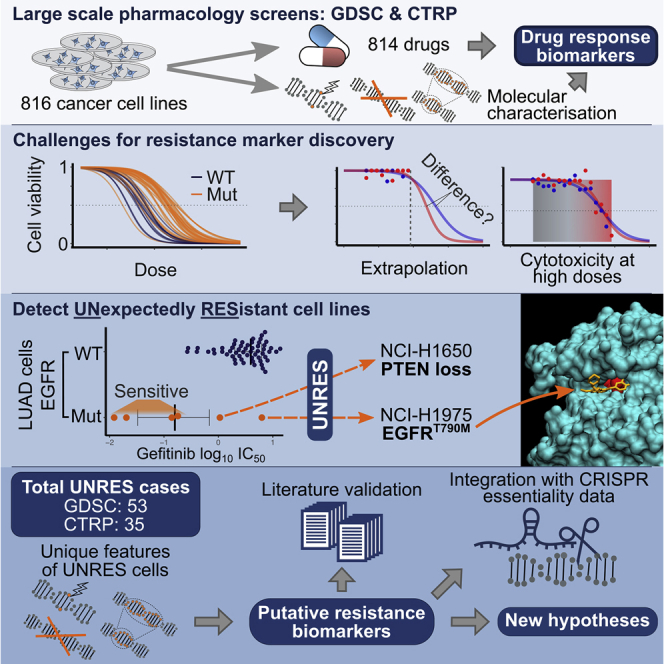

## Teaser

Identifying cancer drug resistance and its biomarkers will empower the next generation of anti-cancer medicines, tailoring treatments to individual patients. Detecting drug resistance in high-throughput pharmacology screens is experimentally challenging. We present a computational framework identifying rare intrinsically resistant cancer cell lines. Our observations provide hypotheses for associated drug-resistance biomarkers, which we validate with independent CRISPR essentiality screens. Our results pave the way for enhancing cancer precision medicine and effective drug combinations to overcome resistance.

## Introduction

Precision medicine has raised high hopes to advance the treatment of cancer.^1^ Treatment with a therapy targeted against an oncogene, i.e., a gene that drives carcinogenesis, places a strong evolutionary pressure on oncogene addicted tumors.^2^ Consequently, subclonal populations in initially responsive tumors can acquire alterations that confer resistance to a given targeted therapy.^3^ Therefore, it is of paramount importance to gain deeper insights into these resistance mechanisms, identify relevant biomarkers, and adjust treatment courses accordingly.^4^

Biomarker discovery is empowered by the high-throughput scalability of cancer cell lines. Following the pioneering work of NCI-60,^5^ which screened 59 cell lines against thousands of compounds, current screening efforts largely expand the cell-line panels to >1,000 cell lines from 30 cancer types for capturing the genetic landscape of cancer. The largest pan-cancer high-throughput screens available are the Genomics of Drug Sensitivity in Cancer (GDSC)^6^^,^^7^ and the Cancer Therapeutics Response Portal (CTRP)^8^, ^9^, ^10^, ^11^ projects (Figure S1A).^12^ These high-throughput screens have been successful in determining drug-sensitivity biomarkers observed in the clinic. For example, *MET* amplifications are associated with sensitivity to savolitinib (an MET inhibitor) in high-throughput screens (Figure S1B), which is currently also under clinical investigation.^13^

Pharmacogenomic screens and models based on systematic statistical inference, pattern matching strategies, and other data-mining methods are capable of identifying drug-resistance biomarkers of frequently mutated cancer genes.^6^^,^^7^ For instance, *TP53* mutants occur in approximately 50% of all samples, and *TP53* mutants in colorectal cancer are associated with nutlin-3a (MDM2 inhibitor) resistance (Figure S1C, S2F, and S2G).^14^ However, many actionable driver gene mutations are infrequent and so missed by the state-of-the-art statistical models. New approaches are required to capture these important events.

**Figure 1:**
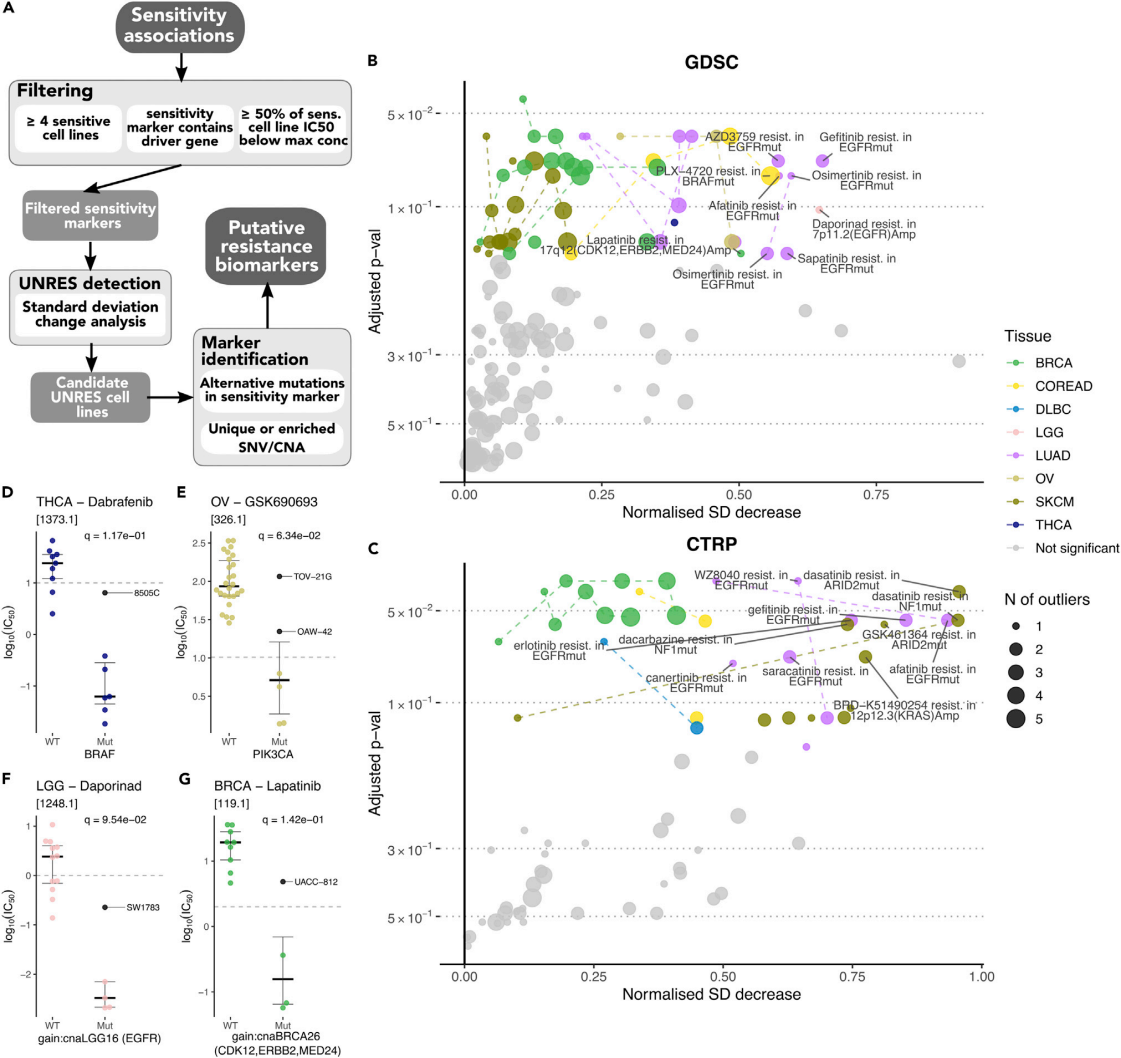
Identification of UNRES Cell Lines (A) Overview of our framework for the identification of resistance biomarkers. (B and C) Result of standard deviation (SD) change analysis for GDSC and CTRP data, respectively. Bootstrap estimates were used to assess significance and corrected for multiple testing to obtain adjusted p values. The magnitude of UNRES cell lines is reported as a normalized measure of decrease in the SD when comparing it with an expected value obtained from the bootstrap distribution. Dashed lines join UNRES cases where different numbers of cell lines were identified as resistant from the same sensitivity association. Details about the specific UNRES cell lines can be found in Tables S5 and S6. Associations with the largest SD decrease (>0.5) are labeled. (D–G) Various examples of identified resistant cell lines (colored black and labeled) and their drug responses in GDSC. q values correspond to adjusted p values as shown in (B). (D) 8505C contains a mutation in *NF2*. (E) TOV-21G contains a *PTEN* mutation among others, whereas OAW-42 shows no marker that could potentially explain resistance. (F) SW1783 contains a *PTEN* mutation. (G) UACC-812 contains a mutation in *CHEK2*, amplifications in 12q15 (*MDM2*, *NUP107*), 20p12.1 (*CRNKL1*, *FOXA2*), 1p12 (*NOTCH2*), and lacks any mutation in *TP53* and any amplification in 17q22 (*CLTC*, *PPM1D*), as opposed to the rest of sensitive cell lines.

An example of secondary resistance to gefitinib (an epidermal growth factor receptor [EGFR] inhibitor) that is frequently observed in lung adenocarcinoma patients, but only infrequently found in cancer cell lines, is the EGFR^T790M^ mutation.^15^ Lung adenocarcinoma cell lines with activating EGFR mutation such as EGFR^L858R^ or exon 19 deletion (Figure S2I), strongly respond to gefitinib.^16^ Unexpectedly, one cell line in GDSC remains entirely resistant (NCI-H1975), which carries the additional EGFR^T790M^ mutation (Figure 1D). Notably, EGFR^T790M^ alters the drug-binding pocket of EGFR, thus preventing the binding of gefitinib and inhibition of EGFR^17^, ^18^, ^19^ (Figure S2J). The identification of this resistance marker led to the approval of gefitinib as first-in-line treatment of patients with non-T790M EGFR mutant metastatic lung cancer, as well as a rapid development of the specific T790M-targeting drug osimertinib.^20^, ^21^, ^22^ This highlights the importance of systematically identifying secondary resistance in preclinical screens to shorten the gap between drug-resistance discovery and patient stratification.

In high-throughput screens, cell lines are commonly treated for a duration of 3–5 days,^6^^,^^7^ reducing the likelihood of observing any resistance mechanism due to acquired alterations. Although these short assays fail to recapitulate acquired resistance, they allow the survey of intrinsic resistance biomarkers, which may share a common molecular basis as the acquired resistance events observed in patients.

The identification of infrequent resistance biomarkers in large pharmacology screens is challenging. Screens such as GDSC were optimized for sensitivity biomarker discovery, resulting in drugs being screened at low concentrations to strictly avoid cytotoxicity, i.e., off-target effects. Therefore, sensitive cell lines are those which respond at low concentrations while resistant cell lines are intermingled with the large bulk of non-responding cell lines (Figures S1B–S1D). We define resistant cell lines as those with an alteration that directly interferes with the drug mode of action, preventing a response even in the presence of a sensitizing event and target activity. In contrast, non-responding cell lines lack a sensitizing event and simply do not respond at given concentrations. Resistant and non-responding cell lines are experimentally indistinguishable.

Drug dose-response curves are typically summarized by the concentration that reduces cell viability by half (IC_50_). It is often the case that the range of tested dose concentrations does not include the IC_50_ of non-responder or resistant cell lines. These values are therefore obtained by curve fitting and extrapolation (Figure S2D).^23^ Screening drugs at higher drug concentrations (e.g., the CTRP approach), may experimentally determine the extrapolated IC_50_ values; however, these still remain challenging to interpret due to cytotoxicity (Figure S2E). Therefore, current methods favor detection of resistance biomarkers with high-frequency altered cancer genes, as these cases putatively have enough statistical power (Figures S1C and S2A–S2C).

To identify infrequent resistance biomarkers in high-throughput drug screens, we present an analysis framework based on the detection of UNexpectedly RESistant (UNRES) cell lines, i.e., cell lines that despite presenting a drug-sensitivity biomarker do not respond to a specific treatment. We integrated pharmacogenomics and CRISPR data from GDSC, CTRP, and the Cancer Dependency Map (DepMap),^24^, ^25^, ^26^ with the available molecular characterization of said UNRES cell lines, to highlight a series of putative resistance biomarkers.

## Results

We employed the biomarker detection framework based on ANOVA models^27^ and the definition of cancer functional events (CFEs) from the GDSC project.^7^ We considered only CFEs involving established cancer driver genes, a minimum of four mutated cell lines, and controlled for observations outside of the concentration range (Experimental Procedures). In total, we accounted for 814 unique drugs and 816 cell lines across GDSC and CTRP (Figure S2H), encompassing a total of 20,238 tested CFE/drug associations for GDSC and 22,173 for CTRP. This resulted in 57 statistically significant (p value < 0.001) cancer-type-specific sensitivity associations with a large and negative signed effect size (Cohen's *d* less than −1) for GDSC (Figure S3A and Table S1) and 37 for CTRP (Figure S3B and Table S2), which are consecutively explored for UNRES cell lines.

### Detection of UNRES Cell Lines

In a first attempt to detect UNRES cell lines, we employed an outlier detection approach using the Neyman-Pearson method^28^ (Supplemental Experimental Procedures; Tables S3 and S4). However, this approach failed to detect clinically established resistance biomarkers, particularly the gold standard that motivates developing this analysis framework, i.e., gefitinib resistance in EGFR^T790M^ mutant in lung adenocarcinoma.

To overcome the limitations of the Neyman-Pearson method, we approached the detection of UNRES cell lines by measuring the standard deviation (SD) of the distribution of drug-response metrics in cell lines with a particular sensitivity biomarker, and observing how much this SD decreases when we ignore the most resistant cell line(s). Applying this UNRES detection pipeline, the GDSC datasets yielded 53 UNRES cases with 1–5 top resistant lines for 23 unique sensitivity associations, and 35 in the CTRP dataset for 22 unique sensitivity associations (adjusted p value < 15%). These correspond to 40.4% and 59.5% of all the sensitivity associations that were analyzed for UNRES presence, respectively. Figures 1B and 1C shows the statistical significance of detected UNRES cell lines (obtained through a bootstrap estimate), along with a normalized metric for the strength of the SD change (see Experimental Procedures) for each case. The names of all the UNRES cell lines identified are summarized in Tables S5 and S6. Figures 1D–1G highlight four examples observed in GDSC with a strong SD decrease.

### Estimation of Mode of Failure

To assess the overall expected number of false positives, we ran 100 repetitions of the full analysis on GDSC data and CTRP where IC_50_ values had been randomly permuted between all cell lines within a tissue (Experimental Procedures and Figure S4). For a range of 3–26 sensitivity associations found in these datasets (Figures S4A and S4B), the average number of significant UNRES cases was 0.96 for GDSC and 0.83 for CTRP, with a range between 0 and 5 in both cases (Figures S4C and S4D). This corresponds to an average of 6.3% and 7.2% of all sensitivity associations detected in the permuted datasets, respectively, as opposed to the observed 40.4% and 59.5%. According to these percentages, we would respectively expect 15.6% and 12.1% of UNRES cases detected in the real dataset to be false positives, which is consistent with our 15% estimate for the UNRES detection level.

The hierarchical nature of our statistical tests (where the first level of testing corresponds to the sensitivity association discovery and the second level to the UNRES detection) adds a layer of interplay between the effects of false positives in both levels, which are not independent. We applied a hierarchical false discovery rate (HFDR) controlling procedure (see Experimental Procedures) that estimates an upper bound of the false discovery rate (FDR) for the entire analysis at the significance levels already stated. This resulted in values of 22.57% and 22.40% for the GDSC and CTRP datasets, respectively.

### Putative Resistance Biomarker Identification

The number of UNRES cell lines is small (Figures S4G and S4H); therefore, we lack statistical power to call resistance biomarkers. However, we can still explore the genomic characterization of cell lines in the GDSC panel based on prior knowledge about cancer biology and mode of action of the drugs. We assembled a list of CFEs unique to (or enriched in) UNRES cell lines, which may become resistance biomarkers (Tables S5 and S6). In summary, we found putative resistance biomarkers for 24 out of the 53 UNRES cases in GDSC and 28 out of 35 in CTRP, ranging between 1 and 9 unique genetic alterations, which may drive resistance.

**Figure 2:**
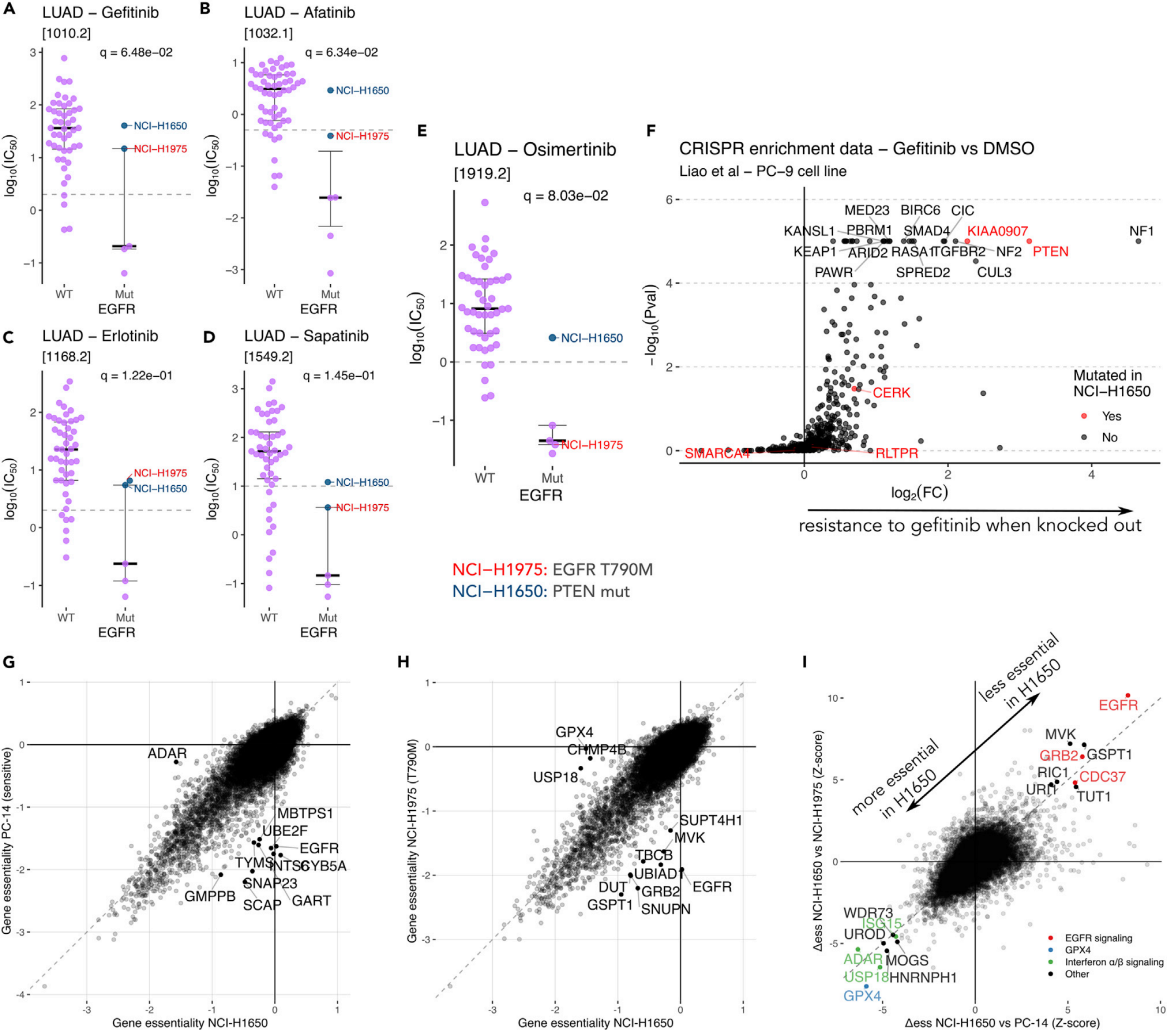
Integration of Identified Hits with Public CRISPR Datasets (A–D) Examples of various EGFR inhibitor responses in GDSC, with two resistant cell lines highlighted: NCI-H1975 (in red), which contains the known EGFR^T790M^ mutation and NCI-H1650 (in blue), which contains an alternative resistance marker, PTEN. q values correspond to adjusted p values as shown in Figure 1B. See also Figures S3C–S3F. (E) Response to an EGFR^T790M^-targeting drug, highlighting the difference between the previously described two resistant cell lines. q value corresponds to the adjusted p value as shown in Figure 1B. See also Figures S3G and S3H. (F) Results from a CRISPR enrichment screen upon gefitinib treatment performed by Liao et al.^30^ in EGFRi-sensitive PC-9 cell lines. Highlighted in red are genes uniquely mutated in NCI-H1650 when compared with the rest of EGFRi-sensitive cell lines. (G and H) Comparison of gene essentiality scores between EGFRi-resistant NCI-H1650 and two other cell lines (EGFR^T790M^ containing NCI-H1975 and classical *EGFR* mutated PC-14). Data were obtained from the Cancer Dependency Map project, and consist of the results of a CRISPR depletion screen in absence of any drug. Negative scores indicate that a certain gene is essential for survival. (I) Joint representation of the differences in essentiality between the two comparisons shown in (G) and (H). We highlight genes for which the behavior in NCI-H1650 is distinct in comparison with both of the other cell lines. See also Figure S6.

We recovered the gold-standard EGFR^T790M^ mutation for gefitinib resistance in *EGFR* mutant lung adenocarcinoma cell lines (Figures 2A and S3D). In addition, we confirmed *PTEN* mutations as putative resistance markers in the *PIK3CA* mutant ovarian serous cystadenocarcinoma cell line TOV-21G, which should have been sensitive to the AKT inhibitor GSK690693 according to its sensitivity biomarker (Figure 1E).^29^

Among the many possible novel resistance biomarkers, we decided to highlight those with high SD decrease magnitudes, as they contain the UNRES cell lines that behave the most differently to the sensitive cell lines. We identified *NF2* mutations associated with resistance to the BRAF inhibitor dabrafenib in *BRAF* mutant thyroid carcinoma cell lines (Figure 1D). This particular cell line (8505C) had been previously identified as resistant to dabrafenib but without a clear associated resistance biomarker.^31^ Other examples include *PTEN* mutation in SW1783 cells associated with resistance to the NAMPT (nicotinamide phosphoribosyltransferase) inhibitor daporinad in *EGFR* amplified low-grade glioma cell lines (Figure 1F). Similarly, we observed that the 17q12 (*ERBB2*) amplified UACC-812 breast cell line is resistant to the ERBB2 inhibitor lapatinib, with some candidate resistance markers being a nonsense mutation in *CHEK2* and amplifications in 2q15 (*MDM2*, *NUP107*), 20p12.1 (*CRNKL1*, *FOXA2*), and 1p12 (*NOTCH2*) (Figure 1G).

We also found a case of clear non-responder (as opposed to resistant) cell lines. In colorectal adenocarcinoma (COREAD), two cell lines (KM12 and LS-513) do not respond to the BRAF inhibitor PLX-4720, despite being *BRAF* mutants (Table S5). The sensitivity biomarker in this case is the specific BRAF^V600E^ mutation, and both of these cell lines contain different *BRAF* mutations, explaining the lack of response.

Focusing on EGFR inhibitor resistance in lung adenocarcinoma cell lines, Figures 2A–2E and S3C–S3F show the EGFR^T790M^ mutant cell line NCI-H1975 as resistant to several EGFR inhibitors. Similarly, NCI-H1650 cell line shows a pattern of resistance similar to that of EGFR inhibitors, even though it lacks the T790M mutation. Most importantly, NCI-H1650 is uniquely resistant to the newest-generation EGFR inhibitor osimertinib, which also targets EGFR^T790M^ mutants.^22^ This is also the case for the EGFR^T790M^-targeting drugs WZ8040 and canertinib, both screened in the CTRP dataset (Figures S3G and S3H).

NCI-H1650 lacks any immediately evident resistance marker, even though our analysis points at 13q34 deletion as a candidate in the case of osimertinib (Table S5) and WZ8040 (Table S6). This deletion is also presented in the EGFR inhibitor-sensitive cell line H3255, which incidentally was not screened with osimertinib, WZ8040, or canertinib. However, NCI-H1650 had previously been described as resistant to EGFR inhibitors due to a homozygous deletion in the 3′ region of *PTEN*. This information is missing from the copy-number alteration data from GDSC, which reports recurrently aberrant copy-number segments identified using ADMIRE.^32^ When inspecting at the gene-level copy number identified based on the PICNIC algorithm,^33^ *PTEN* disruption (and therefore functional loss) is shown to happen. This information is lost when integrating the gene-level copy-number data into recurrently aberrant copy-number segments using ADMIRE (see methods in Iorio et al.^7^), and hence was missing from the annotation we used.

### Integration with Upcoming CRISPR Datasets

CRISPR drug-resistance screens can validate our putative resistance biomarkers. Ideally, we would study whole-genome CRISPR inactivation screens performed on all UNRES cell lines in the presence and absence of the pertinent drug. Such datasets are not yet widespread, but we expect that a robust resistance biomarker could be independently observed in other cell lines from the same cancer type. In the case of loss-of-function mutations, CRISPR depletion screens are an excellent system to validate this. A dataset from Liao et al.^30^ reports gene-enrichment scores in ∼500 cancer-related genes for gefitinib-treated *EGFR* mutant PC-9 cells in comparison with dimethyl sulfoxide (DMSO). Genes with a high enrichment score indicate that their knockout provides selective advantage to the PC-9 cells in the presence of gefitinib, thus their loss of function might be associated with drug resistance. Here we highlight mutations uniquely found in NCI-H1650 (Figure 2F), which is an EGFR inhibitor-resistant cell line. *PTEN* loss is one of the strongest hits, as previously described,^34^ but another strong hit is *KIAA0907*. This gene, also known as *KHDC4*, is involved in pre-mRNA splicing and interacts with the Prp19 complex.^35^ NCI-H1650 contains an N331H missense mutation in an evolutionarily conserved region. There are no further experiments studying the role of this gene in gefitinib resistance, despite being a strong hit in the Liao et al. dataset.^30^

DepMap is another source of CRISPR essentiality experiment data. Differently from the Liao et al. dataset,^30^ the observed phenotype in the DepMap screens is the reduction of viability upon CRISPR/Cas9 targeting of every gene (the gene fitness effect), at a genome-wide (GW) level and across hundreds of cancer cell lines, albeit in the absence of drug treatments. By contrasting the GW profiles of gene fitness effects across cell lines sensitive to a given drug and corresponding UNRES cell lines, we can obtain a set of differentially essential genes (Experimental Procedures), which might shed light on the mechanisms involved in drug resistance as well as potentially targetable vulnerabilities.

Data for the *EGFR* mutants NCI-H1650 (UNRES cell line), NCI-H1975 (EGFR^T790M^ and EGFR^L858R^ mutant), and PC-14 (*EGFR* exon 20 deleted) were available in the DepMap dataset from the Sanger Institute (CERES). Figures 2G and 2H show direct comparisons in essentiality between the UNRES cell line (NCI-H1650) and the other two *EGFR* mutant cell lines. Figures 2I and S6B integrate the differential essentiality ($\text{Δ}ess_{g}$) values across both comparisons, highlighting common differences.

In the absence of treatment, the UNRES cell line (NCI-H1650) does not depend on EGFR signaling for survival, as *EGFR*, *GRB2*, and *CDC37* have high $\text{Δ}ess_{g}$ values >4, indicating that these genes are significantly less essential in this resistant cell line compared with NCI-H1975 and PC-14. This explains the lack of response of NCI-H1650 to EGFR inhibitors, as the cell line is not “addicted” to EGFR signaling. On the other hand, genes with a low (very negative) $\text{Δ}ess_{g}$ value less than −4 are unusually essential in NCI-H1650, which include *GPX4* and the interferon signaling components *ADAR*, *USP18*, and *ISG15*. These genes present vulnerabilities specific to *PTEN-*deficient NCI-H1650 cells. We hypothesize that these vulnerabilities could be exploited in situations where resistance to EGFR inhibitors arises due to the dominance of an alternative signaling pathway such as the PI3K/AKT/mTOR pathway.

As we are only interested in genetic vulnerabilities unique to cancer cells, we also performed differential essentiality analysis comparing NCI-H1650 with *EGFR* wild-type cell lines to filter out vulnerabilities that would also kill healthy tissue. Figure S6A shows a breakdown of Figure 2I separating wt-like genes (genes with essentiality similar to that of the wild-type population) and non-wt-like genes. Most of the identified vulnerabilities are also not present in wild-type cell lines, suggesting that they are unique in resistant cells.

Looking at other UNRES examples, we compared DepMap essentiality scores between GSK690693 resistant and sensitive *PIK3CA* mutant ovarian serous cystadenocarcinoma cell lines (Figure 1E). We performed a separate differential essentiality analysis for each one of the two resistant cell lines, TOV-21G (Figure S7) and OAW-42 (Figure S8), and we have CRISPR data for four sensitive cell lines in this case (OC-314, IGROV-1, OVISE, and OVMIU). The analysis highlights a number of specific genetic vulnerabilities in the resistant cell lines such as *MDM4*, *MYH10*, and *PPP2R1A* in TOV-21G. *PPP2R1A* is a gene encoding for the regulatory subunit of protein phosphatase 2 (PP2A), which is one of the main Ser/Thr phosphatases involved in cell growth and division.^36^ *PPP2R1A* mutations are common across ovarian and endometrial carcinomas,^37^ although this gene has been found to act as a tumor suppressor or a tumor promoter depending on the cellular context.^38^ TOV-21G shows several somatic mutations in genes involved in core signaling pathways such as *PTEN*, *KRAS*, *NF1*, *PIK3R1*, and others (Table S5); therefore, it remains challenging to pinpoint the driver mutation in TOV-21G.

In OAW-42, the other resistant cell line, we can find *NEDD9*, *MBTPS1*, *SCD*, and *SCAP* as unusually essential, among others. *MBTPS1*, *SCD*, and *SCAP* are all involved in regulating cholesterol metabolism,^39^ indicating that OAW-42 might be particularly sensitive to lipotoxicity. Lipogenesis is known to be regulated by the PI3K/AKT/mTOR pathway, and in particular mTORC1 is involved in transcriptional and post-transcriptional regulation of lipogenic enzymes.^40^ We hypothesize that the driver of the resistance to AKT inhibition in this cell line is closely related to mTORC1 signaling in lipid metabolism, which is also supported by the high $\text{Δ}ess_{g}$ values for *TSC1* and *TSC2* (3.98 and 4.76, respectively). Results from the $\text{Δ}ess_{g}$ calculations are fully reported in Table S7.

## Discussion

Our analysis pipeline was able to successfully identify infrequent drug-resistance cell lines and putative biomarkers from large pharmacology screens and validate them with CRISPR screens. Established gold standards with strong evidence in clinics, such as EGFR inhibitor resistance mediated by EGFR^T790M^, were robustly identified by our analysis across both GDSC and CTRP datasets. Most importantly, our framework was capable of systematically identifying infrequent resistance biomarkers from large pharmacology screens, which previously exclusively relied on prior biological knowledge. Our unbiased approach can detect known biomarkers, along with new putative biomarkers, leading the way for hypothesis generation, which complements pooled CRISPR drug-resistance screens. The analysis can be applied to any large pharmacology screen, helping gain insights into resistance mechanisms from new datasets that will be generated in the coming years.

There are two distinct aspects of the analysis that affect its power and introduce some limitations. The first one corresponds to statistical considerations of the UNRES detection. With our HFDR control method, we estimated around 22% of the detected cases to be false positives. A possible pointer for false-positive cases is a small value for normalized SD decrease. These cases typically corresponded to associations where the difference between UNRES cell lines and sensitive ones is similar to the typical difference between any cell line IC_50_s in that tissue, suggesting that any mechanistic difference in drug response is unlikely. Another limitation we observed is that UNRES cell lines with a very low adjusted pvalue “pull” other sensitive cell lines—e.g., the real UNRES case where both NCI-H1975 and NCI-H1650 are resistant to gefitinib was highly significant, but another UNRES case with these two cell lines along with PC-14 (a classical EGFR mutant cell line that is known to be sensitive to gefitinib) was also falsely detected as significant (Table S5).

The second aspect of the analysis that we need to consider is the fact that UNRES groups of cell lines are small, and the identification of putative resistance biomarkers relies heavily on the annotation of each cell line. Inaccurate annotation will inevitably result in inaccurate biomarker discovery. An example of this is the failure in detecting *PTEN* truncation as one of the resistance markers to EGFR inhibitors in NCI-H1650 cell lines. Even with a mostly accurate annotation, the statistical power to compare biomarkers in one resistant cell line versus a handful of sensitive cell lines is very limited. Each cell line has been annotated with hundreds of mutations and copy-number alterations. Thus, when we took a more direct approach by looking for exclusive presence/absence of biomarkers, it yielded many putative results. Many of these are likely to be passengers, requiring further filtering to try to obtain a stronger signal. For this, we used the GDSC cancer gene list, which leverages a large amount of cancer biology knowledge.^7^ This causes an inevitable trade-off between a cleaner signal and a lower sensitivity to detect novel resistance biomarkers previously unrelated to cancer.

In our analysis we focused on genetic biomarkers for drug resistance, however, it is possible that some UNRES cell lines might not have any additional resistance biomarker. For example, some cell lines show no response because they lack the sensitivity marker in the first place (such as the non-V600E BRAF mutant COREAD cell lines, see Results). In other cases, however, resistance might be caused by gene-expression plasticity, epigenetic modifications, or other factors that are outside the scope of this analysis.

The integration of these results with other datasets (i.e., CRISPR essentiality screens) allows for a more unbiased approach, looking at all unique putative markers (not only cancer-related ones) and hinting at effects that may not be exclusively genetic. This is the case for *KIAA0907*, which was not detected as a resistance marker on first instance as it was filtered out with the cancer gene list, but later came up as one of the top hits of the Liao et al.^30^ CRISPR screen for gefitinib resistance. In the Liao et al. study the gene *KIAA0907* remained less explored, since it is not considered as one of the usual suspects in oncology; however, our study builds additional evidence to investigate this gene in more detail.

There is a great opportunity to enhance the results of our analysis pipeline as newer phenotypic datasets become available, in particular CRISPR essentiality screens, both in the presence of a drug of interest (such as Liao et al.^30^) or without any drug (such as results from the Cancer Dependency Map). The integration of pharmacology screens and CRISPR essentiality screens is raising new opportunities to understand drug resistance in the context of the genetic landscape of each cell line.^41^

Furthermore, cell lines identified as resistant and lacking a clear resistance biomarker would be an ideal starting point for a GW CRISPR resistance screen in the presence of the correspondent drug, emphasizing the power of our analysis for hypothesis generation. Notably, our approach is complementary to pooled depletion/activation CRISPR screens, which would not detect secondary resistance mutations such as EGFR^T790M^.

Drug resistance is a clinically important phenomenon that reduces treatment success in cancer patients. Our framework is based on the analysis of large pharmacology screens performed on cancer cell lines, a system that allows high-throughput approaches at the expense of complexity and clinical relevance. However, many relevant insights can be obtained from these models, as proved by the clinically relevant resistance biomarkers we observe in cell lines. Furthermore, it has recently been shown that drug combinations are more likely to be synergistic (up to 20% more) if one of the drugs has a resistance biomarker.^42^ Future work could try to integrate the results of our framework with further methods for the prediction of drug synergy and ultimately pave the way for the next generation of precision medicine.

## Experimental Procedures

### Resource Availability

#### Lead Contact

Michael P. Menden is the lead contact of this study and can be reached by e-mail: michael.menden@helmholtz-muenchen.de.

#### Materials Availability

This study did not generate new unique reagents.

#### Data and Code Availability

All pharmacology data are available at http://www.cancerrxgene.org and https://portals.broadinstitute.org/ctrp.v2.1/. The raw deep molecular characterization is available at https://www.ebi.ac.uk/ega/studies/EGAS00001000978 and https://www.ncbi.nlm.nih.gov/geo/. CRISPR essentiality data were downloaded from https://depmap.org/portal/as the *gene_effect*.*csv* file from the *Sanger CRISPR* (*CERES*) release.

The source code to reproduce all analysis is available at https://github.com/ia327/ayestaran2020_indirect_res, including an interactive Shiny app to explore the generated results.

### Pharmacology Dataset

Cell-line drug-response data were obtained from the GDSC project^6^^,^^7^ and CTRP v2.^9^, ^10^, ^11^ Pharmacology response metric in the GDSC dataset is the drug concentration required to reduce cell viability by half (IC_50_). For the CTRP dataset, IC_50_ values were estimated with the same curve-fitting method used in the GDSC dataset,^23^ as implemented in the R package gdscIC50.^43^ Results of the new curve fitting are reported in Table S8. In total, we analyzed 814 unique drugs in 816 cell lines across 19 cancer types for drug resistance (Figure S2H). From the CTRP dataset, we only included those cell lines also present in GDSC in order to keep molecular characterization data consistent, as described below.

### Cancer Functional Events

Deep molecular characterization of the screened cell lines was obtained from the GDSC project.^7^ CFEs included somatic mutations from whole-exome sequencing, copy-number variations from Affymetrix SNP6.0 arrays, and DNA methylation from IlluminaHumanMethylation450 BeadChip. CFEs are encoded as a binary event for each cell line, being either mutant or wild type. Data processing for variant calling, recurrent altered copy-number segments and informative CpG sites are derived from Iorio et al.^7^

### Drug Sensitivity Association Testing

Biomarkers of drug sensitivity were identified with ANOVA models for each possible combination of cancer type, drug, and CFE as described in Iorio et al.^7^ First, we removed CFEs without established driver genes, i.e., copy-number alterations without known cancer gene, for increasing biological interpretability of results. We enforced a minimum of four mutant cell lines for testing sensitive biomarkers to ensure statistical power for later detecting UNRES cell lines. Finally, we excluded cases where more than 50% of mutant cell lines displayed extrapolated IC_50_ values, as numerical differences between extrapolated data points might be biologically misleading.

We fit an ANOVA model of IC_50_ values to CFE status with the covariates microsatellite instability status, cell-culture medium, and cell-line growth properties. The effect size was estimated with a signed Cohen's *d* statistic,^44^ which for two groups of size $n_{1},\;n_{2}\;$ with means ${\bar{X}}_{1},\;{\bar{X}}_{2}$ and standard deviations ${\text{SD}}_{1},\;{\text{SD}}_{2}$ is defined as

$$d=\frac{{\bar{X}}_{1}-\;{\bar{X}}_{2}}{\sqrt{\frac{\left(n_{1}-1\right)\text{S}{{\text{D}}_{1}}^{2}+\left(n_{2}-1\right)\text{S}{{\text{D}}_{2}}^{2}}{\left(n_{1}+n_{2}-2\right)}}}.$$

A CFE was considered to be a biomarker of drug sensitivity under the conservative threshold p value <0.001 (a p-value filter was chosen, since the GDSC biomarker discovery toolkit^7^ corrects associations by tissue instead of pooled population for FDR, while we used the sensitive population in a posterior hierarchical test) and a signed effect size of less than −1.

### Unexpectedly Resistant Cell-Line Detection

Our primary interests are cell lines derived from the same cancer type carrying a common CFE, which renders this cell population sensitive to a drug, but with distinct UNRES cell lines. We define as UNRES a cell line or group of cell lines that significantly contributes to the sample SD of IC_50_s of a sensitive population. To identify them, we developed an analysis pipeline based on observing changes in the overall SD of a sensitive population when excluding the data point or points with highest IC_50_ values, which are the most resistant cell lines. Let us consider a subpopulation of *n* sensitive cell lines. If we define *σ*_0_ as the SD of the whole set of IC_50_ values, let *σ*_*i*_ be the SD of the distribution when the highest *i* = 1, 2,…, *n*/2 IC_50_ values have been removed (with an upper bound of *i* = 5). The change in SD when removing the highest *i* IC_50_ values will therefore be

$$\text{Δ}\sigma_{i}=\sigma_{i}\;-\sigma_{0}.$$

Significance of each $\text{Δ}\sigma_{i}$ was assessed with a bootstrap method sampling *n* IC_50_ values for that drug and tissue, ignoring CFE status, and calculating the corresponding $\text{Δ}\sigma_{i}\left(\text{boot}\right)$. B=10,000 bootstrap iterations were used, and the p value was defined as

$$p=\frac{1}{B+1}\left(1+\;\sum_{b=1}^{B}\left[\Delta \sigma_{i}\left({\text{boot}}_{b}\right)\;\leq \;\Delta \sigma_{i}\right]\right),$$

where $\left[\Delta \sigma_{i}\left({\text{boot}}_{b}\right)\;\leq \;\Delta \sigma_{i}\right]=1$ if that given bootstrap value is smaller (more negative) than or equal to the observed $\Delta \sigma_{i}$, and 0 otherwise. Multiple testing was corrected using Benjamini-Hochberg FDR correction at level *α* = 0.15.^45^

To quantify the strength of UNRES cases, we additionally calculated a normalized decrease in SD as follows:

$$\text{Normalized SD decrease}=\;-\frac{\text{Δ}\sigma_{i}-E\left[\text{Δ}\sigma_{i}\right]}{\sigma_{0}-E\left[\text{Δ}\sigma_{i}\right]},$$

where $E\left[\text{Δ}\sigma_{i}\right]$ is the expected change in SD when removing the highest *i* IC_50_ values, defined as the median of the bootstrap distribution of $\text{Δ}\sigma_{i}$. This normalized value allows the estimation of the magnitude of the difference between the UNRES cell line(s) and the rest of the sensitive subpopulation, while accounting for the overall spread of IC_50_ values in the corresponding tissue.

### Permutation Test

To obtain an estimate of detected UNRES due to random chance, we performed a permutation test on both datasets. Thus, we randomly permuted the IC_50_ values for each drug within each tissue 100 times and ran our analysis workflows. We summarized the results by counting the number of sensitivity drug-CFE associations, the number of said associations with detected UNRES cases, and the maximum number of UNRES cell lines per association. Significance thresholds were the same as the ones used in the original datasets.

### HFDR Control

To account for the dependence in the hierarchical structure of the statistical tests that identify drug-sensitivity biomarkers with UNRES cell lines, we applied the HFDR controlling procedure developed by Yekutieli.^46^ For this, we arranged the families of hypotheses in two hierarchical levels $L_{0}=\left\{H_{i}:\;\text{sensitivity biomarker}\right\}$ and $L_{1}=\left\{H_{i}:\;\text{UNRES cell lines}\right\}$. All $L_{1}$ hypotheses are associated with a parent hypothesis in $L_{0}$, as described in Figure S5. The employed HFDR approach can be summarized as:

1 Test which parent hypotheses in $L_{0}$ are significant under *α* = 0.001
2 For each significant parent hypothesis, test the hypothesis using the Benjamini-Hochberg method^45^ at level *α* = 0.15 to correct for FDR across all considered $L_{1}$ hypotheses

The parental hypotheses $L_{0}$ were filtered with a conservative p-value threshold in order to guarantee the existence of true drug-sensitivity biomarkers while, under consideration of a large number of tests, sustaining the necessary relaxation level to further investigate UNRES cell lines. Notably, the parental hypotheses with the lowest p values are not necessarily followed by the lowest p values in the child hypotheses, and a simultaneous testing within families was performed as defined by Yekutieli.^46^

Furthermore, to calculate a bound for the overall FDR for all families of hypothesis, we used the approximation

$$\text{FDR}=\left[\frac{\text{No}.\;\text{of}\;\text{discoveries}+\text{No}\;\text{of}\;\text{families}}{\text{No}.\;\text{of}\;\text{discoveries}+1}\right]\times \alpha \times \delta ,$$

where the number of discoveries is defined by the significant markers in $L_{0}$ and $L_{1}$, the number of families are the number of unique combinations of drug and tissue available, and δ is an inflation value close to 1 in most instances,^46^ and therefore assumed equal to 1.

### Resistance Biomarkers

The comparison between identified UNRES cell lines and sensitive populations lacks statistical power because of small sample sizes. Here, we systematically queried the molecular characterization provided by GDSC. For each UNRES cell line, we explored: (1) sensitivity biomarker genes for point mutations that are unique to the UNRES cell line(s); (2) mutually exclusive CFEs: we selected those CFEs that were exclusively mutated in all UNRES cell lines while all sensitive cell lines were wild type, or vice versa; (3) enriched CFEs: in the cases where there were multiple UNRES cell lines, we tested for enriched CFEs with Fisher's exact test between UNRES cell lines and sensitive subpopulation. To narrow down the list of putative markers, we applied a further filter to the identified putative markers by keeping only genes that are included in the GDSC cancer gene list.^7^

### Integration with CRISPR Datasets

Data for the comparison of gene essentialities upon gefitinib treatment was obtained from Liao et al.,^30^ who performed a CRISPR essentiality screen targeting ∼500 tumor-suppressor genes, in the presence of either gefitinib or DMSO. Downloaded gene-enrichment scores upon gefitinib treatment consisted of a fold-change value and its statistical significance, calculated using MaGeCK.^47^

All other CRISPR essentiality data without any treatment were obtained from the DepMap portal (https://depmap.org), specifically the Sanger dataset (processed with CERES).^24^, ^25^, ^26^ The data consist of a matrix of genes × cell lines where each value corresponds to the gene score. High values reflect selective advantage upon knockout of that gene, and low values mean the gene is essential for survival. Note that genes with a low essentiality score (very negative) are considered essential.

Differentially essential genes were selected based on the differences between the essentiality (*ess*) of the gene in the UNRES cell line (*out*) versus a sensitive population of *k* cell lines. For a specific gene *g*, its change in essentiality ($\text{Δ}ess_{g}$) was defined as:

$$\text{Δ}ess_{g}=\frac{1}{k}\sum_{i=1}^{k}\frac{\left(ess_{g}^{out}-ess_{g}^{i}\right)-{\bar{ess}}^{out-i}}{s^{out-i}},$$

where ${\bar{ess}}^{out-i}$ is the sample mean for the raw essentiality differences across all genes and $s^{out-i}$ is the SD of the sample. This method thus obtains a *Z* score for each gene and each comparison between two cell lines and computes the average *Z* score across all comparisons. The mentioned *Z*-transformation is used to account for noisy cell lines before taking the average across all *k* comparisons.

Differential essentiality between the UNRES cell line and wild-type resistant cell lines was calculated with the same method.

## Supplemental Information

Document S1. Supplemental Experimental Procedures and Figures S1–S8

Table S1. Result of Drug-Sensitivity Biomarker Testing with ANOVA Model in GDSC Datasets

Table S2. Result of Drug-Sensitivity Biomarker Testing with ANOVA Model in CTRP Dataset

Table S3. Comparison of UNRES Results for Neyman-Pearson versus Standard Deviation Change Analysis in GDSC Dataset

Table S4. Comparison of UNRES Results for Neyman-Pearson versus Standard Deviation Change Analysis in CTRP Dataset

Table S5. Identified UNRES Cell Lines and Putative Resistance Biomarker in GDSC Datasets

Table S6. Identified UNRES Cell Lines and Putative Resistance Biomarker in CTRP Datasets

Table S7. Differential Essentiality Scores for All the Comparisons between NCI-H1650, TOV-21G, and OAW-42, and Respective Cancer Type Cell Lines

Table S8. Results of Fitting Drug-Response Curves to CTRP Data with the Method Used in GDSC Datasets

Document S2. Article plus Supplemental Information
