# Supplementary material for: Identification of Intrinsic Drug Resistance and Its Biomarkers in High-Throughput Pharmacogenomic and CRISPR Screens
Source: Patterns (N Y). 2020 Jul 2;1(5):100065. doi: 10.1016/j.patter.2020.100065 (PMC7660407; doi:10.1016/j.patter.2020.100065)
Supplement: Document S1. Supplemental Experimental Procedures and Figures S1–S8 [file mmc1.pdf]

PATTER, Volume *1*

Supplemental Information

Identification of Intrinsic Drug Resistance and Its Biomarkers in High-Throughput Pharmacogenomic and CRISPR Screens

Iñigo Ayestaran, Ana Galhoz, Elmar Spiegel, Ben Sidders, Jonathan R. Dry, Frank Dondelinger, Andreas Bender, Ultan McDermott, Francesco Iorio, and Michael P. Menden

SUPPLEMENTAL FIGURES

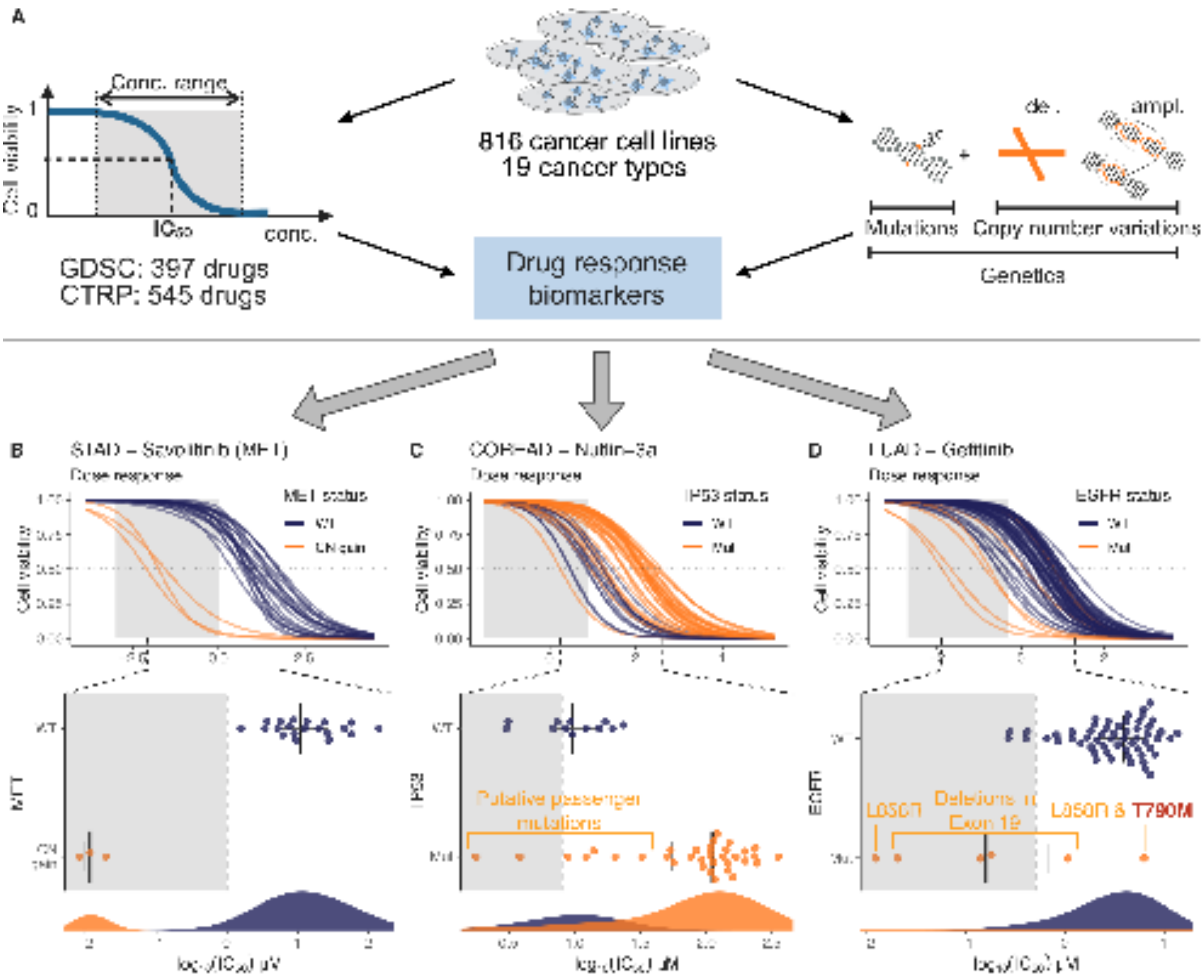

Figure S1: Overview of data and drug response biomarker scenarios. (A) Characteristics of GDSC and CTRP public datasets: drug response data (typically expressed in IC_{50} values) across a collection of 814 unique drugs in 816 cell lines (**Figure S2H**), along with a deep characterisation of point mutations and copy number variations. **(B)** Example of sensitivity biomarker: MET amplification confers sensitivity to the MET inhibitor savolitinib in stomach adenocarcinoma (STAD) cell lines. **(C)** Example of direct resistance biomarker: *TP53* mutations confer resistance to the MDM2 inhibitor Nutlin-3a in colorectal adenocarcinoma (COREAD) cell lines. **(D)** Example of indirect resistance: *EGFR* mutations usually confer sensitivity to the EGFR inhibitor gefitinib, with the exception of a resistant outlier that contains the secondary rare resistance biomarker $EGFR^{T790M}$.

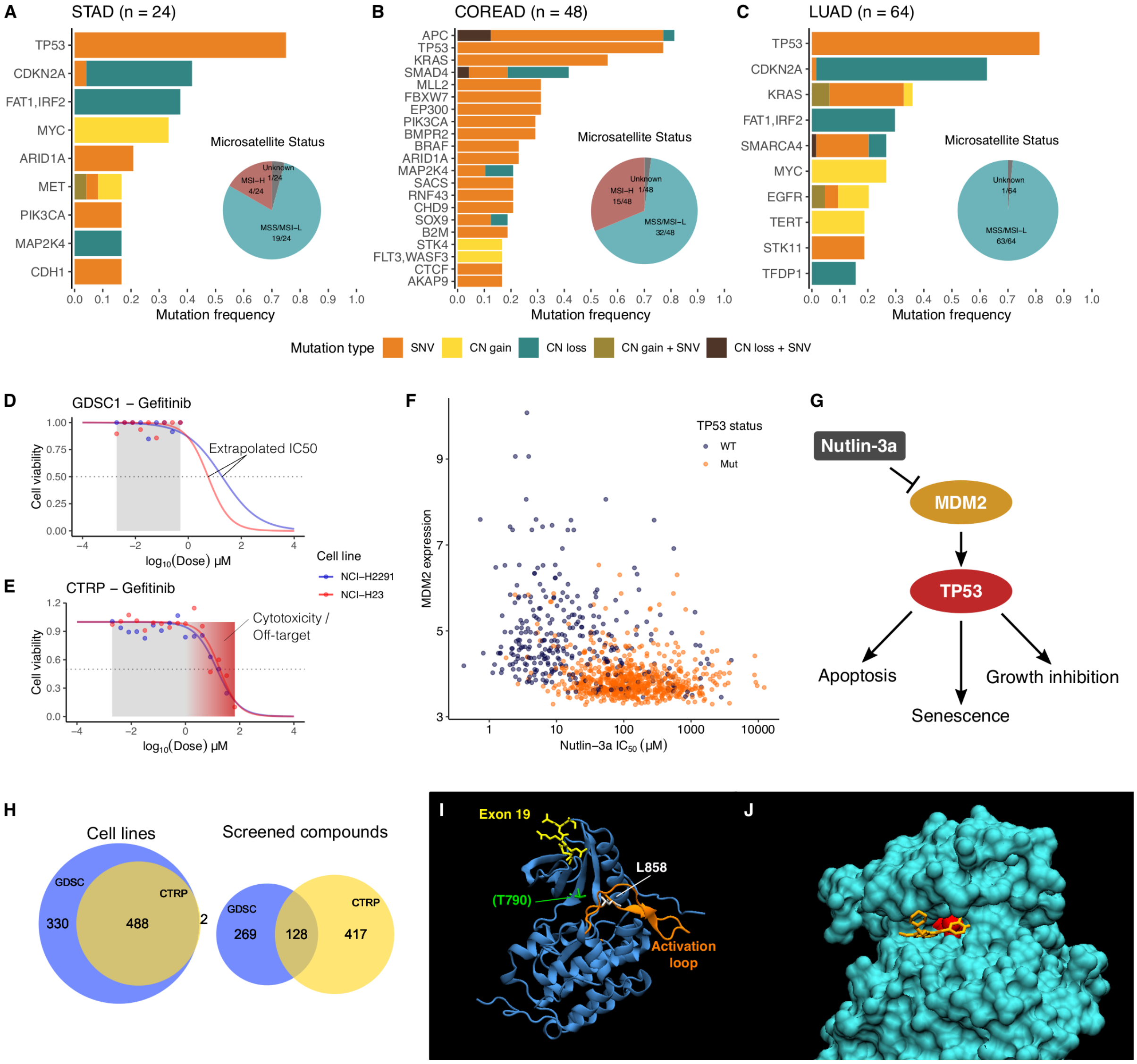

Figure S2. Synopsis of pharmacology screens and response examples. (A-C) Frequencies of the most common mutations in GDSC cell lines for stomach adenocarcinoma (STAD), colorectal adenocarcinoma (COREAD) and lung adenocarcinoma (LUAD). Inset shows the proportions of microsatellite stable or low instability (MSS/MSI-L) cell lines and cell lines with microsatellite instability (MSI-H). **(D)** Example of the raw viability data from GDSC for 2 cell lines treated with gefitinib and the response curve fitted. IC50 values are extrapolated when cells are resistant/non-respondent. **(E)** Example of the raw viability data from CTRP for 2 cell lines treated with gefitinib and the response curve fitted. The dose range is bigger than GDSC, but high concentrations can result in cytotoxic or off-target effects. **(F)** Correlation between *TP53* status, *MDM2* expression and sensitivity (IC50) to nutlin 3a. **(G)** Diagram of the effect of nutlin 3a on the cell with relation to *TP53* and *MDM2*. **(H)** Venn diagrams with total numbers and overlaps of cell lines and screened compounds used in this study from GDSC and CTRP. **(I)** 3D structure of EGFR (PDB ID: 1M17, Stamos et al, 2002) with the most common oncogenic mutations highlighted. Brackets around T790 indicate that the mutation in this residue is not oncogenic per se, but generates drug resistance. **(J)** Detail of the drug binding pocket of EGFR with T790 highlighted in red and gefitinib in yellow (PDB ID: 2ITY, Yun et al, 2007). Structures were rendered using VMD (Humphrey et al, 1996).

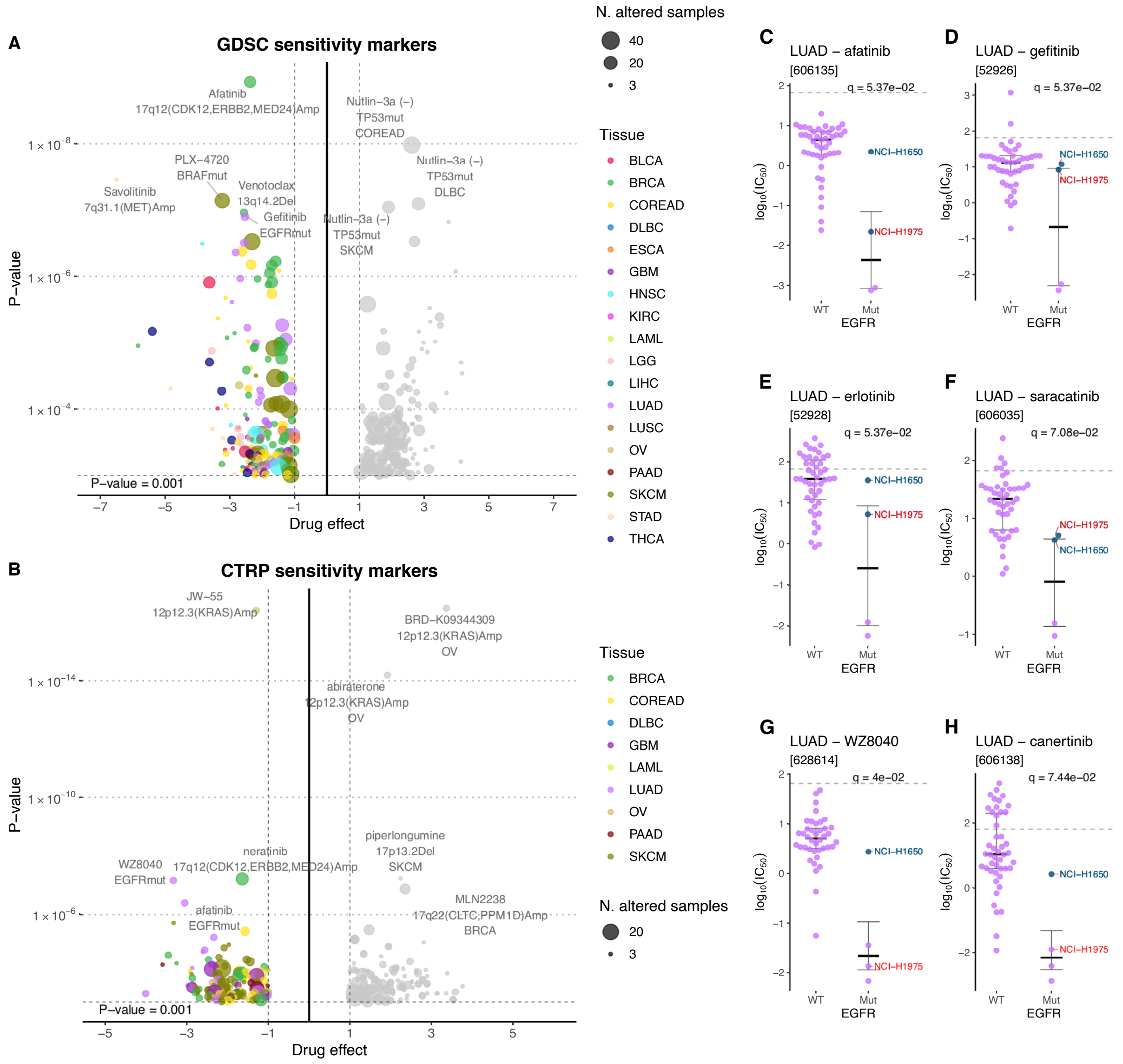

Figure S3. Results for sensitivity biomarker discovery and CTRP UNRES identification. (A-B) Results from a systematic ANOVA test for differential drug response in GDSC and CTRP, using Cancer Functional Events as factors. Points on the left side of the plot represent drug sensitivity markers. **(C-F)** Examples of various EGFR inhibitor responses with 2 resistant cell lines highlighted: NCI-H1975 (in red), which contains the known $EGFR^{T790M}$ mutation and NCI-H1650 (in blue) which contains an alternative resistance marker, PTEN. Related to **Figure 2A-D** **(G-H)** NCI-H1650 shows resistance to specifically T790M targeting drugs WZ8040 and canertinib, while NCI-H1975 is sensitive. Related to **Figure 2E**.

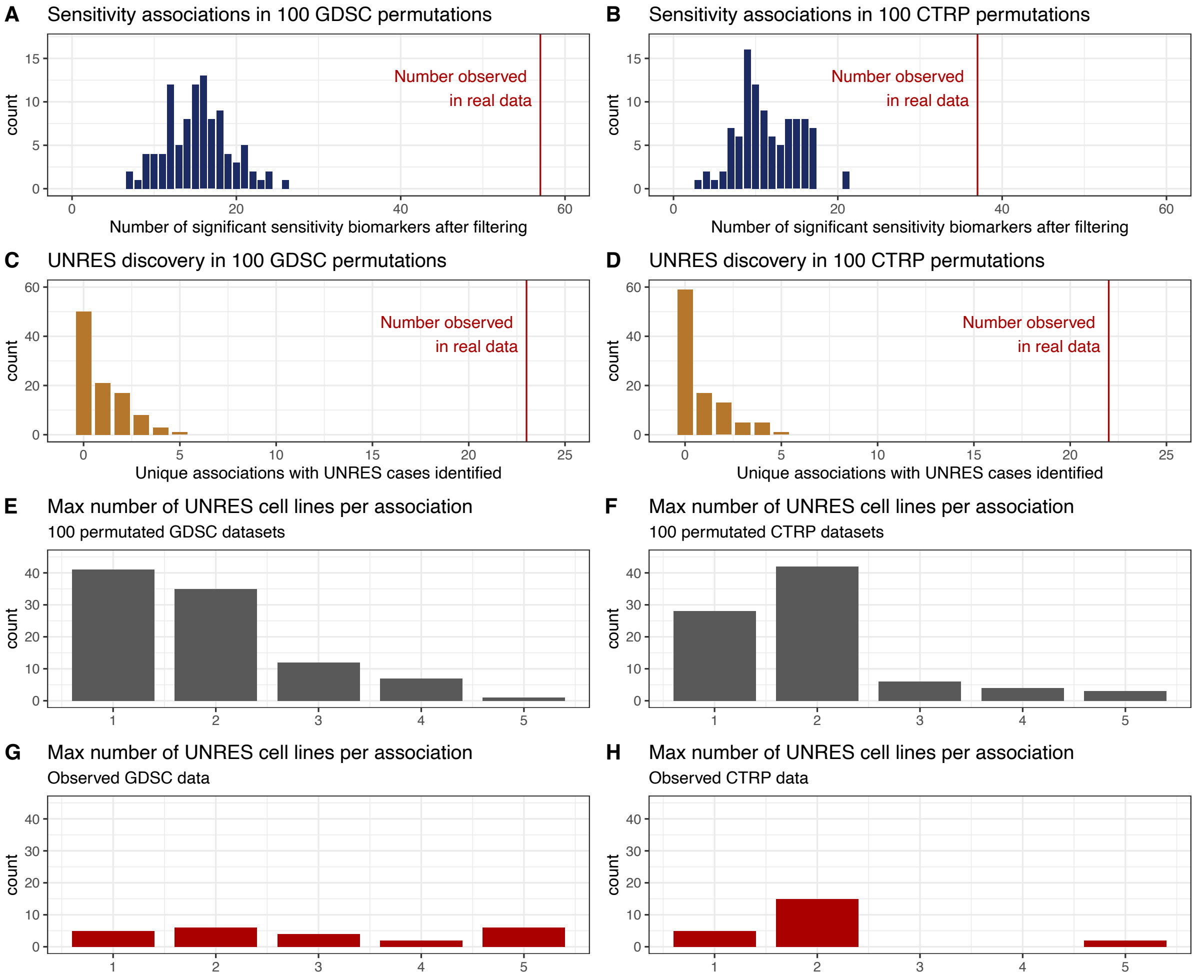

Figure S4: Summary of permutation tests for estimation of false discoveries. (A-B) Histogram showing the number of statistically significant ($p<0.001$) drug sensitivity associations detected in 100 permuted datasets. The red line shows the numbers detected in original datasets. **(C-D)** For the same permuted datasets, number of UNRES cases detected (p-adjusted<0.15) Red lines show the numbers detected in original datasets. **(E-F)** Maximum number of cell lines detected as UNRES for each sensitivity association in 100 permuted datasets. **(G-H)** Maximum number of cell lines detected as UNRES for each sensitivity association in original data.

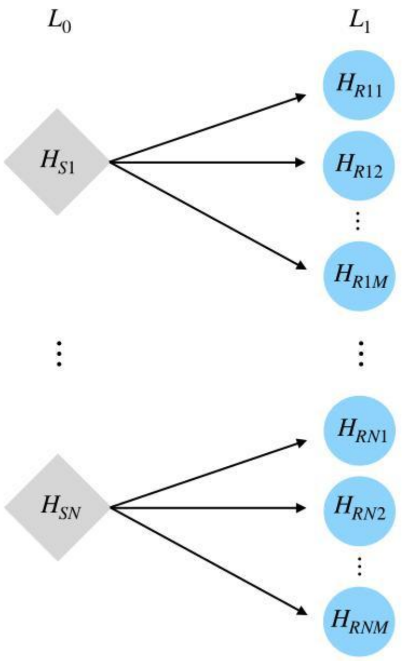

Figure S5: Hierarchical FDR procedure illustration. A two level hierarchical tree schematic with level-0 and level-1 defined by $L_0 = \{H_{Si}, i = 1, \ldots, N\}$ and $L_1 = \{H_{Rij}, i = 1, \ldots, N; j = 1, \ldots, M\}$, respectively. Level-0 hypothesis H_{Si} tests whether a given cell line is a sensitive biomarker, whilst level-1 hypothesis H_{Rij} tests, under the rejection of parent hypothesis H_{Si}, if the sensitive cell with the j-th highest IC_{50} removed is a resistance biomarker.

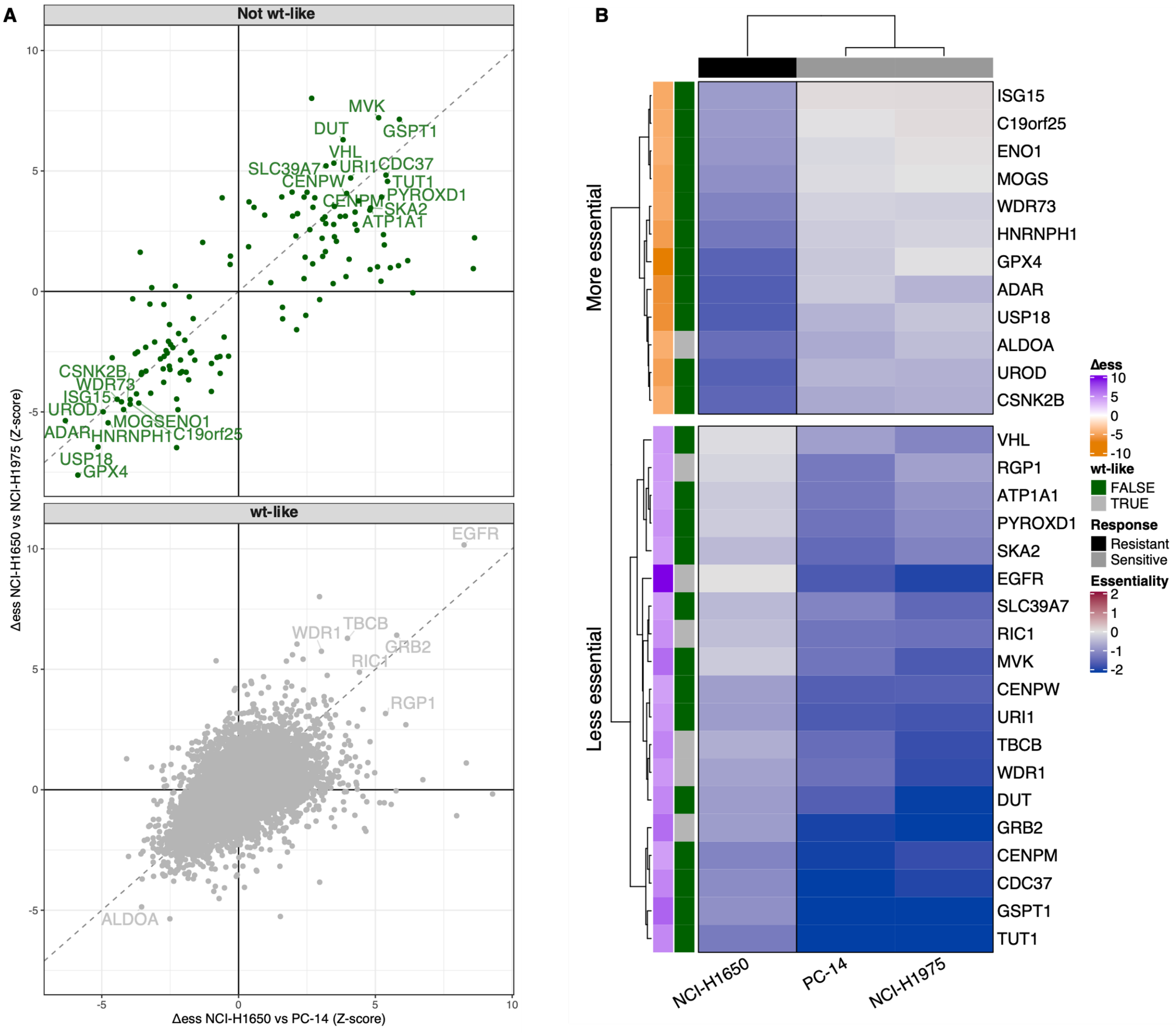

Figure S6: Differential essentiality for NCI-H1650 from DepMap data. Related to Figure 2I. (A) Equivalent to Figure 2I but with genes split into 2 panels according to the differential essentiality vs *EGFR* wild-type cell lines. Genes were defined as wt-like if $|\Delta ess_g| < 3$. **(B)** Heatmap summarising genes with a highest Δess_g across both comparisons. Genes were selected if $|\Delta ess_g| > 4$ and the |Z-score| for each comparison < 3. The essentiality of genes related to EGFR signalling behaves like in EGFR wild type cell lines. Most potential unique vulnerabilities of NCI-H1650 (bottom left corner in **A**, top half in **B**) are also different to wild type cell lines.

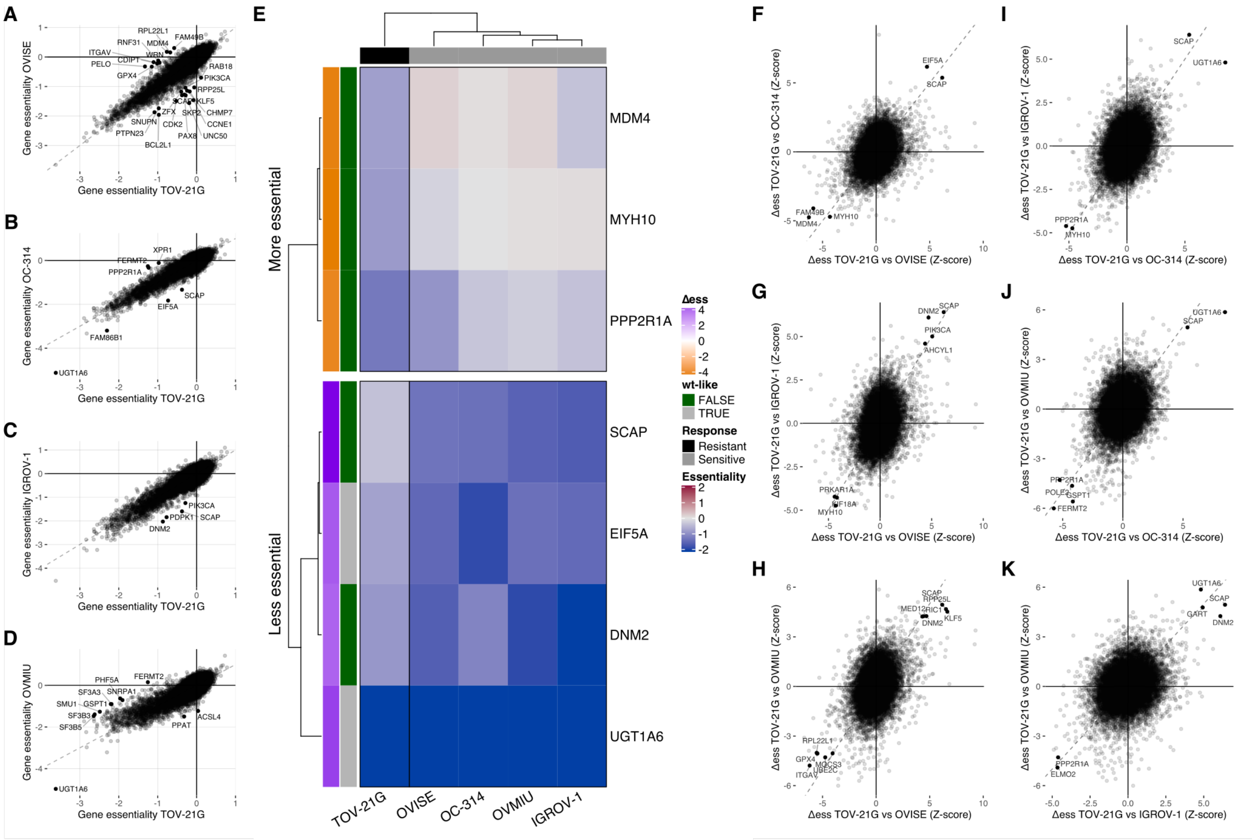

Figure S7: Comparison of DepMap essentiality data for TOV-21G vs other OV cell lines. (A-D) Direct comparisons of gene essentialities between one resistant cell line and GSK690693 sensitive cell lines. Negative values represent deleterious knock-outs (essential genes) and high positive values indicate advantageous knock-outs. **(E)** Heatmap summarising genes that are most differentially essential across all comparisons. Genes were selected if $|\Delta ess_g| < 4$ and the |Z-score|< 3 for at least 2 of the comparisons. **(F-K)** A score for the difference between the resistant and one cell line is calculated (Δess) and Z-normalised. Each pairwise comparison of Δess_g values is represented, highlighting genes that are commonly differentially essential ($|\Delta ess_g| < 4$ in both comparisons).

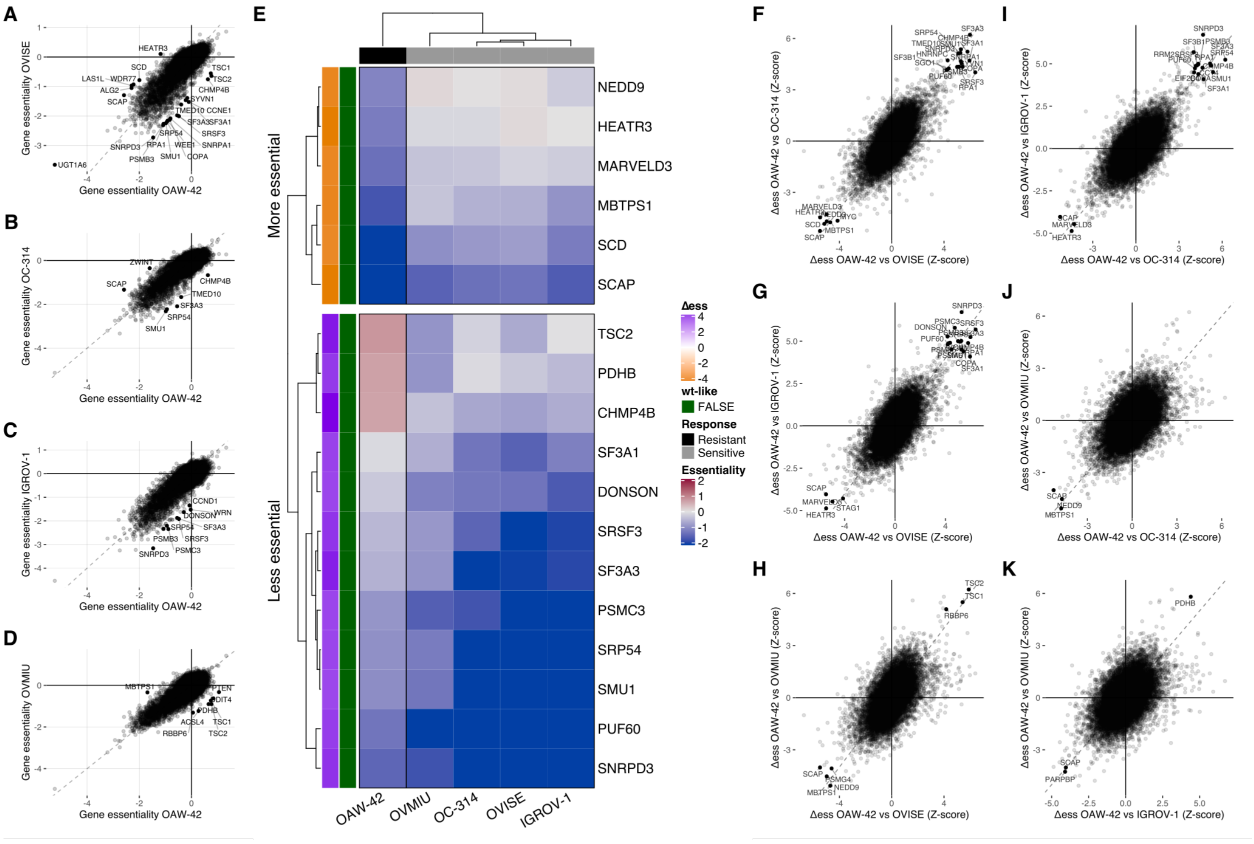

Figure S8: Comparison of DepMap essentiality data for OAW-42 vs other OV cell lines. See legend of **Figure S7**.

SUPPLEMENTAL EXPERIMENTAL PROCEDURES

Comparison to an outlier detection method

In an attempt to identify an unexpectedly resistant subpopulation of cell lines with sensitivity biomarker, we adopted the multiple hypothesis test Neyman-Pearson (NP)[1] approach by considering the detection of UNRES cells as a statistical hypothesis test. For this, we define the distribution of IC_{50} values (X_i) of all associations of drug, tissue and mutated CFE status of sensitive cells as $X \sim N(\mu_0, \sigma_0)$, and address our objective as the hypothesis test:

$$H_0 : X < x_{critical}$$
$$H_1 : X \geq x_{critical}$$

The critical region is defined, according to the critical value $x_{critical}$, as the upper tail of the distribution (where one would expect to detect resistant cell lines) under significant level α=0.15.

SUPPLEMENTAL REFERENCES

1. Neyman, J., Pearson, E.S., Pearson, K. (1933). IX. On the problem of the most efficient tests of statistical hypotheses. Philosophical Transactions of the Royal Society of London. Series A, Containing Papers of a Mathematical or Physical Character 231, 289–337. 10.1098/rsta.1933.0009.
